# Ischemic preconditioning attenuates ischemia/reperfusion injury in rat steatotic liver: role of heme oxygenase-1-mediated autophagy

**DOI:** 10.18632/oncotarget.13281

**Published:** 2016-11-10

**Authors:** Anding Liu, Enshuang Guo, Jiankun Yang, Renlong Li, Yan Yang, Shenpei Liu, Jifa Hu, Xiaojing Jiang, Olaf Dirsch, Uta Dahmen, Jian Sun, Mingwen Ouyang

**Affiliations:** ^1^ Experimental Medicine Center, Tongji Hospital, Tongji Medical College, Huazhong University of Science and Technology, Wuhan, China; ^2^ Experimental Transplantation Surgery, Department of General, Visceral and Vascular Surgery, Friedrich-Schiller-University Jena, Jena, Germany; ^3^ Department of Infectious Diseases, Wuhan General Hospital of Guangzhou Military Command, Wuhan, China; ^4^ Department of Biliopancreatic Surgery, Sun Yat-sen Memorial Hospital, Sun Yat-sen University, Guangzhou, China; ^5^ Department of Anesthesiology, The Fifth Affiliated Hospital, Southern Medical University, Guangzhou, China

**Keywords:** ischemic preconditioning, steatosis, liver ischemia/reperfusion injury, autophagy, heme oxygenase-1, Pathology Section

## Abstract

Steatotic livers are more susceptible to ischemia/reperfusion (I/R) injury, which is ameliorated by ischemic preconditioning (IPC). Autophagy possesses protective action on liver I/R injury and declines in steatotic livers. The aim of this study was to test the hypothesis that the increased susceptibility of steatotic livers to I/R injury was associated with defective hepatic autophagy, which could be restored by IPC via heme oxygenase-1 (HO-1) signaling. Obesity and hepatic steatosis was induced using a high fat diet. Obesity impaired hepatic autophagy activity and decreased hepatic HO-1 expression. Induction of HO-1 restored autophagy activity and inhibited calpain 2 activity. Additionally, suppression of calpain 2 activity also restored autophagy activity. Mitochondrial dysfunction and hepatocellular injury were significantly increased in steatotic livers compared to lean livers in response to I/R injury. This increase in sensitivity to I/R injury was associated with defective hepatic autophagy activity in steatotic livers. IPC increased autophagy and reduced mitochondrial dysfunction and hepatocellular damage in steatotic livers following I/R injury. Furthermore, IPC increased HO-1 expression. Inhibition of HO-1 decreased the IPC-induced autophagy, increased calpain 2 activity and diminished the protective effect of IPC against I/R injury. Inhibition of calpain 2 restored autophagic defect and attenuated mitochondrial dysfunction in steatotic livers after I/R. Collectively, IPC might ameliorate steatotic liver damage and restore mitochondrial function via HO-1-mediated autophagy.

## INTRODUCTION

Ischemia/reperfusion (I/R) injury is a major cause of liver injury during hepatic surgery [1, 2]. Hepatic steatosis is one of the most common hepatic disorders in developed countries. It is known that steatotic livers are particularly vulnerable to I/R injury [3–6]. Ischemic preconditioning (IPC) is defined as a brief period of ischemia followed by a short interval of reperfusion prior to prolonged period of ischemia. IPC has been proven to be an effective surgical strategy to reduce liver I/R injury in experimental models [7–10]. Its beneficial effects in clinical liver surgery are controversial [11–14]. The discrepancy may be explained by the concept that the benefit of IPC is proportional to the severity of I/R injury [11]. In the majority of clinical studies, IPC appears to decrease liver damage in patients with hepatic steatosis [15–18]. The precise mechanisms by which IPC confers protection to liver I/R injury are not yet fully understood.

Autophagy is an evolutionarily conserved cellular process which degrades and recycles damaged proteins and organelles via the lysosomal degradation and is considered as an adaptive response to stress [19, 20]. Specifically, mitochondrial autophagy (or mitophagy), which binds and removes damaged and dysfunctional mitochondria, is a key cellular process that regulates mitochondrial homeostasis and prevents cell death in liver I/R injury [21]. Autophagy has also been reported to be linked to lipid metabolism and is impaired in steatotic livers [22–24]. Furthermore, autophagy plays a protective role in liver I/R injury, and enhancement of autophagy may represent a novel therapeutic strategy to ameliorate liver I/R injury [25–28].

IPC restores autophagy activity in human steatotic liver grafts, reduces liver damage and [18]. We have demonstrated that IPC protects against I/R Injury via induction of autophagy in lean livers [29]. However, the contribution of the autophagic mechanism to the IPC-afforded protection in steatotic livers and the potential mechanisms involved are still not fully understood.

Heme oxygenase-1 (HO-1), a stress-responsive enzyme, is highly induced during oxidative stress response including I/R situation. Recently, HO-1 has been shown to protect against liver I/R injury via induction of autophagy [30], and HO-1-mediated autophagy is an essential element of protection conferred by IPC [29]. However, how does HO-1 increase autophagy activity has not yet been elucidated. Earlier studies have shown that calpain 2 hydrolyzes autophagy proteins [26, 31]. Furthermore, a recent study suggests that the impaired autophagy in steatotic livers may result from an increase in calpain 2 activity [24]. Thus it is reasonable to regard that IPC could prevent calpain 2-impaired autophagy by increasing HO-1.

The aim of this study was to investigate the role of autophagy in IPC-afforded protection on steatotic livers, as well as the regulatory mechanisms of autophagy, particularly its linkage to the HO-1 system.

## RESULTS

### Autophagy is impaired in steatotic livers

**Figure 1:**
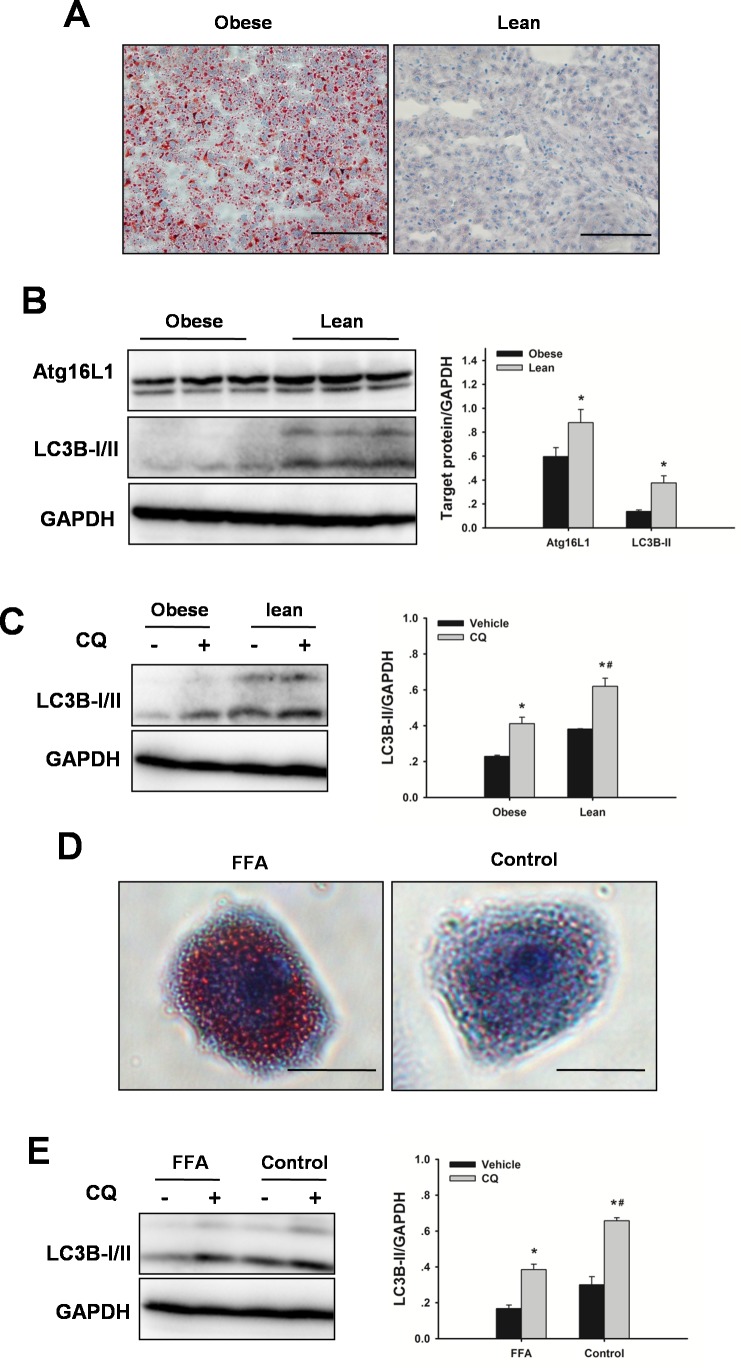
Obesity decreases hepatic autophagy Rats received a HFD for 8 weeks to induce obesity. **A.** Representative Oil Red O staining micrographs displaying liver sections. Original magnification x 400, scale bars 100 μm. **B.** Autophagic proteins Atg16L1 and LC3B expression in liver were examined by western blot analysis. The data are shown as the mean ± SD. *n* = 6 per group. *p < 0.05 compared to the lean group. **C.** To determine autophagic flux, rats received chloroquine (CQ, 60 mg/kg, IP) 6 h before sacrifice. Western blot analysis of LC3B protein expression in the presence and absence of CQ. The data are shown as the mean ± SD. *n* = 6 per group. *p < 0.05 compared to the vehicle group, #*p* < 0.05 compared to the obese + CQ group. **D.** Hepatocytes were cultured with FFA (1 mM, 2: 1 ratio of oleate: palmitate) for 24 h. Oil Red O staining of lipids in hepatocytes. Original magnification x 400, scale bars 50 μm. **E.** For autophagic flux, hepatocytes were treated with 10 μM CQ for 6 h before harvest. Western blot analysis of LC3B protein expression in the presence and absence of CQ. The experiment was performed in triplicates with similar results. *p < 0.05 compared to the vehicle group, #*p* < 0.05 compared to the FFA + CQ group.

To investigate the regulation of autophagy in steatotic livers, western blots were performed for on steatotic livers and lean livers. Obese rats have more lipid accumulation in liver compared to age-matched lean animals (Figure 1A). Obesity resulted in markedly decreased autophagy indicators in liver, as indicated by down-regulation of autophagy-related protein (Atg) 16L1 and microtubule-associated protein 1 light chain (LC) 3B-II protein levels compared with the lean controls (Figure 1B). Furthermore, lean livers displayed a robust autophagy activity, as shown by a marked increase in LC3B-II expression by chloroquine. In contrast, obesity impaired autophagic flux, as shown by lower expression levels of LC3B-II in steatotic livers by chloroquine (Figure 1C). A similar finding was observed in vitro. Steatotic hepatocytes (free fatty acids (FFA)-induced) exhibited a marked reduction of autophagic flux compared with the hepatocytes without FFA treatment (Figure 1D, 1E). These findings confirmed that hepatic autophagy activity was down-regulated in obesity.

### Impaired autophagy in steatotic livers is mediated by HO-1/calpain 2 signaling

**Figure 2:**
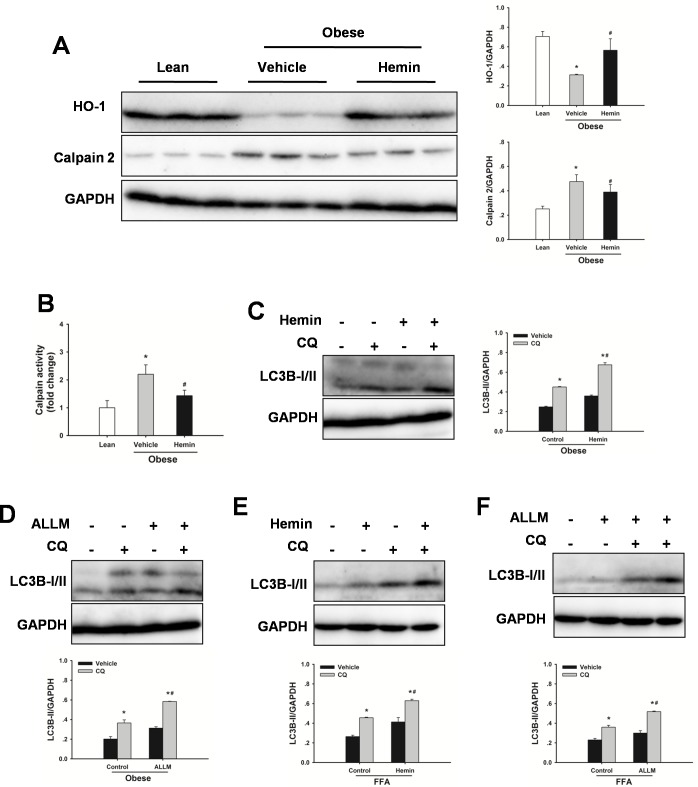
Defective autophagy is mediated by HO-1/calpain 2 signaling **A.** Obese rats were administered with hemin (30 mg/kg, IP) or vehicle three times a week for 2 weeks. Expression of HO-1 and calpain 2 protein was determined by western blot analysis. **B.** Measurement of calpain activity in liver. The data are shown as the mean ± SD. *n* = 6 per group. *p < 0.05 compared to the lean group, #*p* < 0.05 compared to the vehicle group. **C.** Obese rats were treated with hemin, western blot analysis of LC3B protein expression in the presence and absence of chloroquine (CQ, 60 mg/kg, IP). **D.** Obese rats were treated with ALLM (10 mg/kg, IP), western blot analysis of LC3B protein expression in the presence and absence of CQ. **E.** Changes in autophagic flux in steatotic hepatocytes with hemin (50μM). **F.** Changes in autophagic flux in steatotic hepatocytes with ALLM (50μM). The experiment was performed in triplicates with similar results. The data are shown as the mean ± SD. *p < 0.05 compared to the vehicle group, #*p* < 0.05 compared to the control + CQ group.

HO-1 is thought to be a protective response from cellular stress and a key mediator of autophagy [29, 30, 32]. Hence, HO-1 protein expression levels in livers were investigated. As shown in Figure 2A, HO-1 expression levels were decreased in steatotic livers compared to lean controls. Treatment with hemin significantly increased HO-1 protein expression levels. Furthermore, Hemin significantly increased autophagic flux both in steatotic livers in vivo and in FFA-treated hepatocytes in vitro (Figure 2C, 2E). To evaluate the contribution of calpain 2 to the impaired autophagy, calpain 2 protein expression levels in steatotic livers were examined. As shown in Figure 2A and 2B, obesity markedly increased calpain 2 protein expression levels and increased calpain activity in liver compared with those in the lean control, demonstrating that calpain 2 was activated in steatotic livers. Treatment with hemin significantly prevented the increase in calpain 2 protein expression levels and calpain activity, indicating an important role of HO-1 in calpain activation. N-acetyl-leucyl-leucyl-methioninal (ALLM), a membrane-permeable calpain inhibitor, suppresses calpain 2 activation, but not prevent calpain 1 activity [33]. ALLM treatment restored autophagic flux both in livers from obese rats in vivo and in FFA-treated hepatocytes in vitro (Figure 2D, 2F). These results indicate that obesity-induced decrease in HO-1 activity may be an important mechanism leading to the defective autophagy.

### Impaired autophagy contributes to the increased sensitivity of steatotic livers to I/R injury

**Figure 3:**
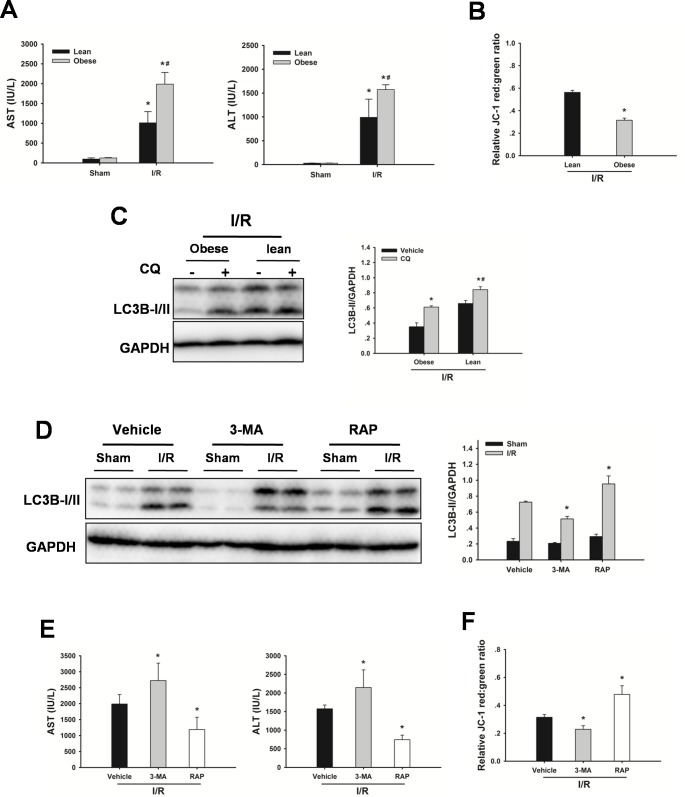
Defective autophagy increases liver I/R injury **A.** Obese rats or lean rats were subjected to 60 min ischemia and 6 h reperfusion. AST and ALT levels were analyzed as a measure of hepatocellular injury. Data are shown as the mean ± SD, *n* = 6 per group. *p < 0.05 compared to the sham group, #*p* < 0.05 compared to the lean + I/R group. **B.** MMP was measured by JC-1 fluorescence red: green ratio. The data are shown as the mean ± SD. *n* = 6 per group. *p < 0.05 compared to the lean + I/R group. **C.** Western blot analysis of LC3B protein expression in the presence and absence of chloroquine (CQ, 60 mg/kg, IP). *p < 0.05 compared to the vehicle + I/R group, #*p*< 0.05 compared to the obese + CQ + I/R group. **D.** Obese rats were pretreated with 3-methyladenine (3-MA, 30 mg/kg, IP), or rapamycin (RAP, 1 mg/kg, IV) 60 min prior to warm ischemia. Autophagic protein LC3B in the ischemic lobes was examined by western blot analysis. **E.** AST and ALT levels. (B) MMP was measured by JC-1 fluorescence red: green ratio. The data are shown as the mean ± SD. *n* = 6 per group. *p < 0.05 compared to the vehicle + I/R group.

As shown in Figure 3A, following 60 min of warm ischemia and 6 h reperfusion, serum aspartate aminotransferase (AST) and alanine transaminase (ALT) levels were significantly increased compared with those in sham rats. In contrast, the serum levels of AST and ALT were significantly higher in obese rats than those in age-matched lean animals. I/R caused a decrease in mitochondrial membrane potential (MMP). The MMP in steatotic livers was lower than those in lean livers, demonstrating obesity led to an increased mitochondrial dysfunction in response to I/R injury (Figure 3B). To determine whether impaired autophagy could contribute to the increased sensitivity to I/R injury, autophagic flux firstly was examined in steatotic livers subjected I/R injury. Autophagic flux in steatotic livers was lower than those in lean controls following I/R injury (Figure 3C). To determine the role of autophagy in liver I/R injury, autophagy was suppressed by use of the established pharmacological inhibitor 3-methyladenine (3-MA), or induced by rapamycin. As shown in Figure 3D-3F, western blot analysis revealed 3-MA treatment reduced -II expression following I/R injury, indicating that autophagy was inhibited. Furthermore, treatment with 3-MA augmented I/R-induced increases in serum AST and ALT levels and decrease in MMP. Conversely, western blot analysis revealed rapamycin treatment increased-II expression, indicating that autophagy was activated. Rapamycin treatment decreased the increase in AST and ALT levels, and prevented the decrease in MMP in response to I/R insult. These results indicated that the increased sensitivity of steatotic livers to I/R injury may result from the defect in autophagy activity.

### IPC protects steatotic livers from I/R injury via induction of autophagy

**Figure 4:**
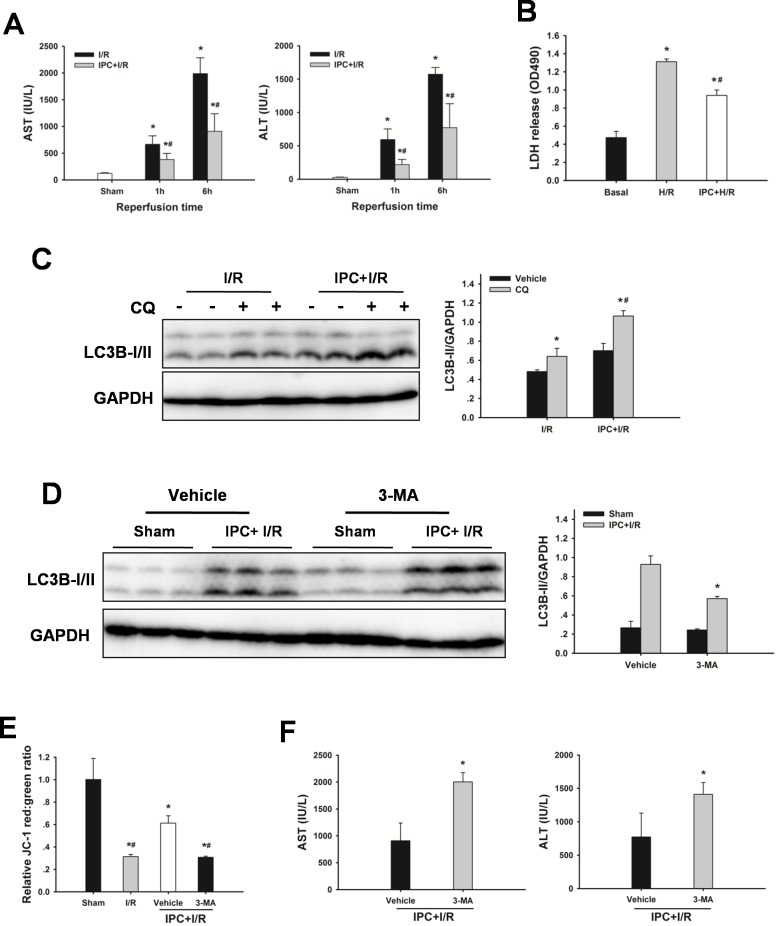
IPC restores autophagic flux in steatotic livers following I/R injury **A.** Obese rats were subjected to 10 min of ischemia followed by 10 min reperfusion prior to 60 min warm ischemia. AST and AST levels. The data are shown as mean ± SD. *n* = 6 per group. *p < 0.05 compared to the sham group, #*p* < 0.05 compared to the I/R group. **B.** Steatotic hepatocytes were subjected to 10 min of ischemia followed by 10 min reperfusion prior to H/R insult. Quantification of medium LDH levels. The experiment was performed in triplicates with similar results. The data are shown as the mean ± SD. *p < 0.05 compared to the basal group, #*p* < 0.05 compared to the H/R group. **C.** Changes in autophagic flux in steatotic livers with or without IPC. The data are shown as the mean ± SD. *n* = 6 per group. *p < 0.05 compared to the vehicle group, #*p* < 0.05 compared to the CQ + I/R group. **D.** Rats were pretreated with 3-methyladenine (3-MA, 30 mg/kg, IP) 30 min prior to IPC. Autophagic protein LC3B in the ischemic lobes was examined by western blot analysis following reperfusion. The data are shown as the mean ± SD. *n* = 6 per group. *p < 0.05 compared to the vehicle + IPC + I/R group. **E.** Mitochondrial membrane potential was measured by JC-1 fluorescence red: green ratio. The data are shown as the mean ± SD. *n* = 6 per group. *p < 0.05 compared to the sham group, #*p* < 0.05 compared to the vehicle + IPC + I/R group. **F.** ALT and AST levels were analyzed as a measure of hepatocellular injury. The data are shown as the mean ± SD. *n* = 6 per group. *p < 0.05 compared to the vehicle + IPC + I/R group.

Hepatic I/R significantly increased serum AST and ALT levels, which were significantly decreased by IPC. The serum levels of AST and ALT were drastically decreased following reperfusion in the IPC group, AST from 1988 ± 295 to 908 ± 329 IU/L, and ALT from1573 ± 101 to 773 ± 356 IU/L at 6 h after reperfusion, respectively (Figure 4A). In vitro, IPC also resulted in a decreased cell death following hypoxia/reoxygenation (H/R) injury, as measured by medium lactate dehydrogenase (LDH) levels (Figure 4B).

**Figure 5:**
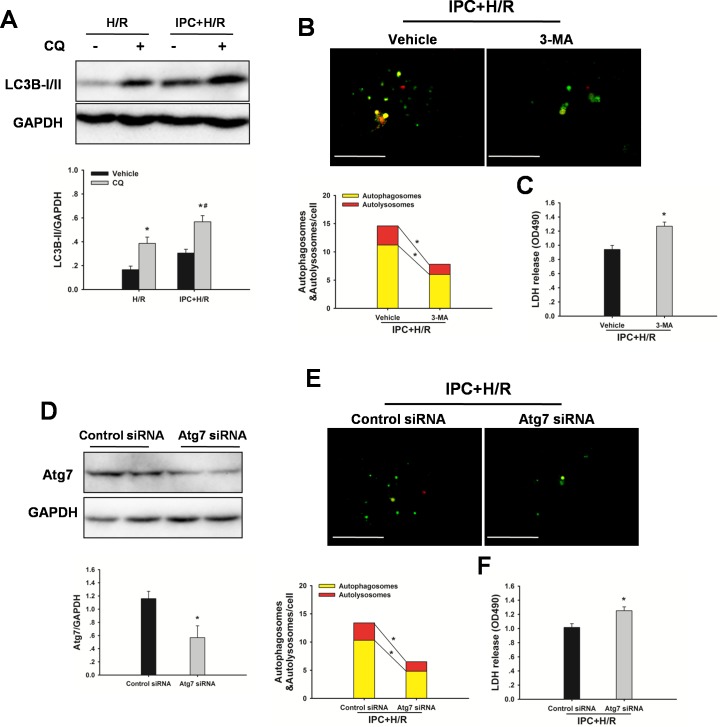
IPC induces autophagic flux following H/R insult Steatotic hepatocytes were subjected to 10 min of ischemia followed by 10 min reperfusion prior to hypoxia. **A.**Western blot analysis of LC3B protein expression in the presence and absence of chloroquine (CQ). *p < 0.05 compared to the vehicle group, #*p* < 0.05 compared to the CQ + H/R group. **B.** Hepatocytes, infected with adenovirus encoding mRFP-GFP-LC3, were subjected to IPC+ H/R in the presence or absence of 3-MA. Representative images of fluorescent LC3 puncta are shown. Original magnification x 400, scale bars 50 μm. Quantification of the number of autophagosomes represented by yellow dots in merged images and autolysosomes represented by red dots in merged images. *p < 0.05. **C.** Quantification of cultured medium LDH levels. *p < 0.05 compared to the vehicle + IPC + H/R group. **D.** Immunoblots indicating expression of Atg7 protein after treatment with HO-1 siRNA for 48 h. *p < 0.05 compared to the control siRNA group. **E.** Representative images of fluorescent LC3 puncta are shown. Original magnification x 400, scale bars 50 μm. Quantification of the number of autophagosomes represented by yellow dots in merged images and autolysosomes represented by red dots in merged images. *p < 0.05. **F.** Quantification of cultured medium LDH levels. *p < 0.05 compared to the control siRNA + IPC + H/R group. The experiment was performed in triplicates with similar results. The data are shown as the mean ± SD.

To determine whether IPC could attenuate steatotic liver I/R injury via inducing autophagy, the effect of autophagy on the IPC-afforded protection was investigated. As shown in Figure 4C, increased -II levels, and administration of chloroquine led to further increases of LC3-II levels, indicating a strong autophagic response to reperfusion. Furthermore, treatment with 3-MA attenuated the IPC-induced LC3-II expression, decreased MMP and abolished the protection conferred by IPC (Figure 4D-4F). In vitro, IPC also markedly increased autophagic flux in steatotic hepatocytes. Inhibition of IPC-induced autophagy by 3-MA or Atg7 small interfering RNA (siRNA) increased cell death, respectively (Figure 5).

### IPC-induced autophagy is dependent on HO-1/calpain 2 signaling

**Figure 6:**
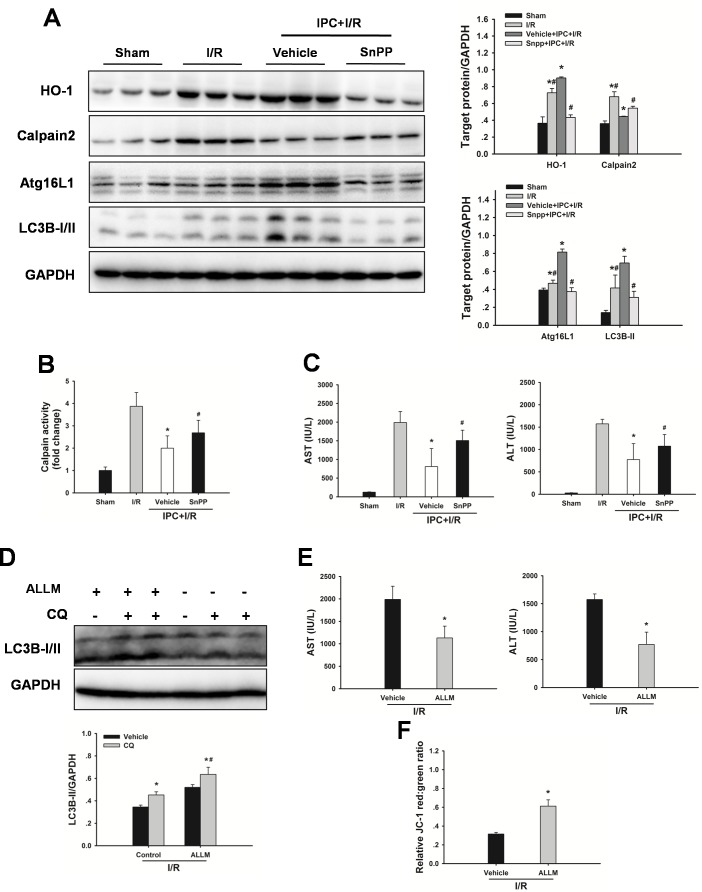
Inhibition of HO-1 by SnPP attenuates the IPC-induced hepatoprotection **A.** Obese rats were pretreated with SnPP (50 mg/kg) 1 h prior to IPC. HO-1, calpain 2, Atg16L1 and LC3B expression in the ischemic lobes was examined by western blot analysis. The data are shown as the mean ± SD. *n* = 6 per group. *p < 0.05 compared to the sham group, #*p* < 0.05 compared to the vehicle + IPC + I/R group. **B.** Measurement of calpain activity. **C.** Serum AST and ALT levels were analyzed as a measure of hepatocellular injury. The data are shown as the mean ± SD. *n* = 6 per group. *p < 0.05 compared to the I/R group, #*p* < 0.05 compared to the vehicle + IPC + I/R group. **D.** Western blot analysis of LC3B protein expression in the presence and absence of chloroquine (CQ). The data are shown as the mean ± SD. *n* = 6 per group. *p < 0.05 compared to the vehicle group, #*p* < 0.05 compared to the CQ + I/R control group. **E.** Serum AST and ALT levels were analyzed as a measure of hepatocellular injury. **F.** Mitochondrial membrane potential was measured by JC-1 fluorescence red: green ratio. The data are shown as the mean ± SD. *n* = 6 per group. *p < 0.05 compared to the vehicle + I/R group.

**Figure 7:**
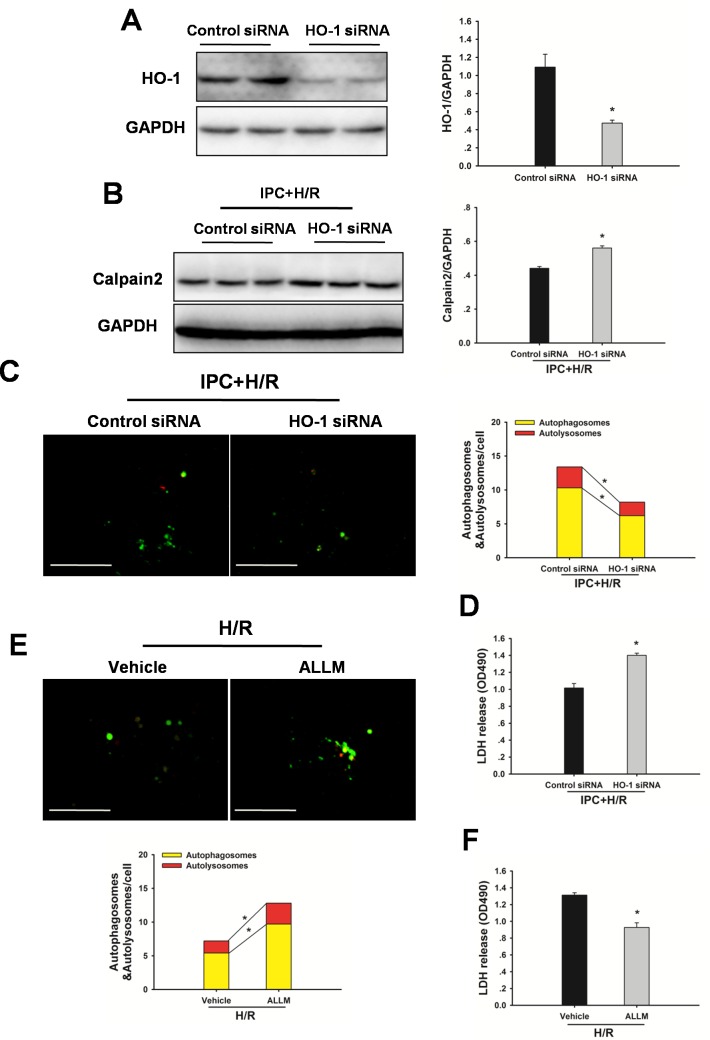
Inhibition of HO-1 by HO-1 siRNA attenuates the IPC-induced hepatoprotection in vitro **A.** Immunoblots indicating expression of HO-1 protein after treatment with HO-1 siRNA for 48 h. *p < 0.05 compared to the control siRNA group. **B.** Western blot analysis of calpain 2 protein expression. *p < 0.05 compared to the control siRNA + IPC + H/R group. **C.** Representative images of fluorescent LC3 puncta are shown. Original magnification x 400, scale bars 50 μm. Quantification of the number of autophagosomes represented by yellow dots in merged images and autolysosomes represented by red dots in merged images. *p < 0.05. **D.** Quantification of cultured medium LDH levels. *p < 0.05 compared to the control siRNA + IPC + H/R group. **E.** Steatotic hepatocytes were subjected to H/R in the presence and absence of ALLM. Autophagy was determined by mRFP-GFP-LC3 analysis. *p < 0.05. **F.** Quantification of cultured medium LDH levels. *p < 0.05 compared to the vehicle + H/R group. The experiment was performed in triplicates with similar results. The data are shown as the mean ± SD.

To evaluate the possible autophagy signaling pathway through which IPC exerts its regulatory action on autophagy, HO-1 signaling was examined. As shown in Figure 6A, hepatic HO-1 protein expression levels were increased in steatotic livers with IPC in response to I/R injury. In addition, calpain 2 protein levels and calpain activity were decreased in the IPC group. Inhibition of HO-1 activity using SnPP abolished the IPC-mediated decrease in calpain 2 activity (Figure 6B). Furthermore, IPC-induced LC3-II and Atg16L1 protein expression levels were significantly attenuated by SnPP treatment (Figure 6A). Importantly, SnPP treatment abolished the hepatoprotection afforded by IPC, as indicated by marked increases of AST and ALT levels (Figure 6C). Additionally, ALLM treatment restored autophagic flux, attenuated I/R-induced increases in serum ALT and AST levels, and blocked decrease in MMP (Figure 6D-6F). In vitro, Inhibition of HO-1 activity using HO-1 siRNA significantly prevented the IPC-induced increase in autophagy, reduction in calpain 2 activity and decrease in LDH level. Furthermore, ALLM significantly increased autophagy and prevented cell damage in response to H/R insult (Figure 7).

## DISCUSSION

**Figure 8:**
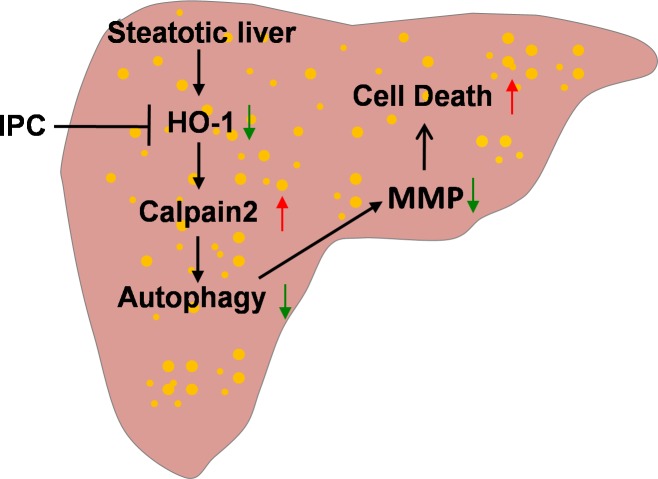
Scheme of possible protective mechanism of IPC on steatotic liver I/R injury Obesity decreases hepatic HO-1, which in turn increases calpain 2 activity. Calpain 2 hydrolyzes autophagy proteins, subsequently impairs autophagy activity and leads to MMP decrease and cell death. IPC increases hepatic HO-1, which restores defective hepatic autophagy via blocking calpain 2 activity. Restoration of autophagy prevents MMP decrease and cell death.

We previously demonstrated that autophagy was induced by IPC, and played a pivotal role in protecting against I/R injury in lean livers [29]. However, little attention has been given to autophagy in IPC-mediated protection in steatotic liver I/R injury, and the exact underlying mechanisms remain unclear. In this study, our results demonstrated that: (1) obesity subsequently impaired hepatic autophagy activity, which was mediated by HO-1/calpain 2 signaling; (2) this impairment of autophagy activity contributed to the greater vulnerability to I/R insult; and (3) IPC restored the calpain 2-impaired autophagy via induction of HO-1, thereby inhibited mitochondrial dysfunction and protected steatotic liver from I/R injury (Figure 8).

Autophagy is involved in lipid metabolism [22–24]. Under physiological conditions, autophagy (lipophagy) participates in the basal turnover of lipids by engulfing and degrading lipid droplets. Autophagy is impaired in steatotic livers and inhibition of autophagy increases hepatic lipid storage during starvation [22], which corroborates our finding of a decreased autophagy activity both in steatotic livers in vivo and in FFA-treated hepatocytes in vitro. This decline of autophagy activity has been linked to various mechanisms, such as over-activation of mammalian target of rapamycin (mTOR), a defect in lysosomal acidification, a reduction in cathepsin L, and a defect in autophagosome-lysosome fusion [34]. Recently, Yang and colleagues [24] demonstrated that an obesity-induced increase in calcium-dependent protease calpain 2 activity led to degradation of Atg7 then to a defective autophagy. However, how does obesity enhance the activity of calpain 2 remains unclear. Recently, we and others have demonstrated that HO-1 plays an important role on regulation of autophagy [29, 30, 32]. Therefore, it is reasonable to assume that the defective autophagy may result from an obesity-induced decrease in HO-1. In the present study, HO-1 expression was significantly decreased, whereas calpain 2 activity was significantly increased in steatotic livers. Induction of HO-1 significantly reduced calpain 2 activity and increased autophagy flux. Furthermore, inhibition of calpain 2 activity effectively reversed the defective autopahgy flux in steatotic livers. These findings were confirmed in vitro studies. Taken together, these results provide evidence that an obestiy-induced decrease in HO-1 may contribute to the defective autophagy in steatotic livers.

There is evidence that steatotic livers are more susceptible to I/R injury [3–6]. However, the exact underlying mechanisms have not been fully understood. Autophagy, a protective mechanism in liver I/R injury, is impaired in steatotic livers [22–24]. It is plausible that the increased susceptibility to I/R injury may due to the defective autophagy, therefore induction of autophagy may ameliorate I/R injury in steatotic livers. These notions have been supported by our observations. We demonstrated that mitochondrial dysfunction and liver damage were significantly increased, whereas autophagic flux was substantially impaired in steatotic livers in response to I/R injury. We found that inhibition of autophagy by 3-MA significantly reduced autophagy and worsened I/R injury. In contrast, induction of autophagy by rapamycin significantly increased autophagy, attenuated mitochondrial dysfunction and liver I/R injury in steatotic livers. These data suggest that the increased susceptibility of steatotic livers to I/R injury is associated with depletion of autophagy activity.

IPC is able to attenuate I/R injury in steatotic livers both in experimental animals [7–10] and in clinical trials [15–18]. Consistent with these observations, we demonstrated that IPC reduced I/R injury. More recently, increasing evidence supports that the protective effect of IPC is associated with its ability to enhance autophagy [18, 29]. In this study, we showed the IPC increased autophagy activity in steatotic livers in response to I/R injury. To confirm the role of autophagy in the IPC-afforded protection, the IPC-induced autophagy was blocked by pretreatment with 3-MA. As expected, 3-MA abolished the IPC-afforded protection against I/R injury in steatotic livers. These results imply that autophagy may be an important pathway in the IPC-afforded protection in steatotic liver I/R injury.

We previously demonstrated that the IPC-induced autophagy was mediated by HO-1 signaling [29]. In the current study, we showed that HO-1 expression was decreased in steatotic livers, and the impaired autophagy may result from the depletion of HO-1. Therefore, it is possible that IPC could protect against I/R injury via HO-1-mediated autophagy. As expected, IPC increased HO-1 expression. Inhibition of the IPC-induced increase in HO-1 reduced autophagy flux and worsened liver injury. Furthermore, HO-1 inhibition eliminated the IPC-induced decrease in calpain 2 activity, which is directly associated with autophagy defect after I/R [26, 35]. These findings were supported by Zhao et al, who demonstrated that calpain 2-mediated autophagy defect increased susceptibility of fatty livers to I/R injury[36]. These data support that the IPC-induced autophagy is mediated, at least in part, by HO-1/calpain 2 signaling in steatotic livers.

In conclusion, obesity impairs autophagy activity, which is mediated by HO-1 inhibition and consequent calpain 2 activation. The defective autophagy further contributes to the increased sensitivity of steatotic livers to liver I/R injury. IPC attenuate liver I/R injury in steatotic livers, and the protective mechanism appeared to involve its ability to induce autophagy via HO-1.

## MATERIALS AND METHODS

### Experimental design

A diet-induced obesity rat model (high fat diet, HFD, 8 weeks) was used in this study. To investigate whether obesity could impair autophagy activity in livers via HO-1/calpain 2 signaling, hemin (30 mg/kg, intraperitoneal [IP], Sigma-Aldrich, St. Louis, MO), or ALLM (10 mg/kg, IP, Cayman Chemical, Ann Arbor, MI) was administered three times a week for 2 weeks to induce HO-1 or inhibit calpain 2 activity, respectively. To measure autophagic flux, chloroquine (60 mg/kg, IP, Sigma-Aldrich) was administered 6 h before sacrifice. In vitro, steatotic hepatocytes ((FFA)-induced) were treated with hemin (50 μM) or ALLM (50 μM) 24 h before harvest. For autophagic flux, steatotic hepatocytes were treated with 10 μM chloroquine.

To determine whether the increased susceptibility of steatotic livers to I/R injury was associated with the defect in hepatic autophagy activity, rats were pretreated with rapamycin (1 mg/kg, intravenous [IV], Sigma-Aldrich) to induce autophagy, or 3-MA (30 mg/kg, IP, Cayman Chemical) to inhibit autophagy 1 h prior to warm ischemia, respectively. Rats were sacrificed at 6 h of reperfusion, and liver injury and autophagic flux were analyzed.

To investigate whether IPC could protect liver I/R injury via HO-1-induced autophagy, 3-MA was given to rats 30 min prior to ischemia to inhibit IPC-induced autophagy. HO activity was inhibited in vivo through an injection of tin protoporphyrin IX (SnPP, 50 mg/kg, IP, Santa Cruz Biotechnology, Santa Cruz, CA) 30 min prior to ischemia. Calpain 2 was blocked by ALLM 1 h prior to ischemia. Rats were sacrificed at 6 h of reperfusion, and liver injury and autophagic flux were analyzed.

To investigate whether IPC could protect steatotic hepatocytes from H/R injury via HO-1-induced autophagy in vitro, autophagy was inhibited by 3-MA (10 mM) or Atg7 siRNA (50 nM, Life Technologies, Carlsbad, CA), and HO-1 was suppressed by HO-1 siRNA (50 nM, Life Technologies) prior to hypoxia, respectively. Calpain 2 was blocked by ALLM. Hepatocytes were harvested after 6 h hypoxia and 2 h reoxygenation. Hepatocellular injury and autophagic flux were analyzed.

### Experiential animals

All experimental procedures were performed in accordance with the National Institutes of Health guide for the care and use of laboratory animals approved by the ethical committee for the use of experimental animals at Huazhong University of Science and Technology (SYXK-2014-0049). Male inbred Sprague-Dawley rats (weighing within 160~180g) were housed under standard animal care conditions and allowed free access to food and water. Some of rats received a HFD for 8 weeks to develop fatty livers.

### Liver I/R injury model

A model of segmental (70%) hepatic ischemia was performed as described previously [37]. Briefly, rats were completely anesthetized by pentobarbital (60 mg/kg, IP). A midline laparotomy was performed and a micro vascular clamp was used to interrupt blood supply to the hepatic arterial and portal venous branches to the left lateral and median liver lobes for 60 min. As controls, sham operation was performed by anesthesia and laparotomy only. IPC was produced by 10 min of ischemia and 10 min of reperfusion prior to ischemic insult.

### Cell cultures, steatosis and H/R

Primary rat hepatocytes were isolated and cultured by a modified in situ collagenase perfusion technique as described [32, 38]. The viability of hepatocytes was 90% as determined by trypan blue exclusion. The hepatocytes were cultured on plates coated with rat tail collagen (Sigma-Aldrich) in Dulbecco's Modified Eagle's Medium (DMEM, Life Technologies) supplemented with 10 % fetal bovine serum. To induce the steatosis, hepatocytes were incubated in DMEM medium containing FFA at a final concentration of 1 mM (2: 1 ratio of oleate: palmitate, Sigma-Aldrich) for 24 h to induce fat overloading [39]. Steatotic hepatocytes were placed in a hypoxia chamber for 6 h in hypoxic media, followed by 2 h reperfusion (media with 10% serum). The IPC protocol was determined at 10 min of hypoxia followed by 10 min of reoxygenation.

### siRNA transfection

Atg7 siRNA, HO-1 siRNA, or scrambled siRNA was transfected using Lipofectamine RNAiMAX reagent (Life Technologies) following the manufacturer's guidance. After transfection for 48 h, hepatocytes were treated with different conditions for further analysis.

### mRFP-GFP-LC3 adenovirus transfection

Hepatocytes were infected with adenovirus encoding mRFP-GFP-LC3 (20 multiplicity of infection, Hanbio, Shanghai, China). The number of autophagosomes (red and green foci) and autolysosomes (red-only foci) were calculated for evaluation of autophagic activity under an inverted Olympus FV1000 laser scanning confocal microscope (Olympus, Tokyo, Japan).

### Hepatocellular damage assessment

Hepatocellular damage was assessed in vivo by measuring serum AST and ALT levels using an automated chemical analyzer (Hitachi Co, Tokyo, Japan), and in vitro by measuring medium LDH using a colorimetric LDH cytotoxicity assay kit (BioVision, Milpitas, CA) according to the manufacturer's protocol.

### Oil Red O staining

Steatosis in liver tissue and primary hepatocyte was evaluated using Oil Red O staining according to standard procedures.

### Gel electrophoresis and western blotting

Liver tissues or cultured cells were homogenized as described previously [40]. Equal amounts of protein were separated on 12 % gels by sodium dodecyl sulfate-polyacrylamide gel electrophoresis and transferred to polyvinyldifluoride membranes. The membranes were incubated overnight at 4°C with primary antibodies: rabbit anti-HO-1(1:1000, Abcam, Cambridge, UK), rabbit anti-LC3B (1:1000, Abcam), rabbit anti-Atg16L1(1:1000, Abgent, San Diego, CA), rabbit anti-calpain2 (1:500, Cell Signaling Technology, Beverly, MA), rabbit anti-Atg7 (1:500, Cell Signaling Technology) and rabbit anti-glyceraldehyde-3-phosphate dehydrogenase (GAPDH) antibody (1:20000; Sigma-Aldrich). Immunoreactive band signals were developed using ECL system (GE Healthcare, Buckinghamshire, UK) and visualized on the Kodak Image Station (Carestream Health Inc, New York, USA). Relative band densities were quantified using the Image J software (NIH, Bethesda, USA).

### Calpain activity assay

Calpain activity was determined using calpain activity assay kit (Abcam) according to the manufacturer's instructions.

### Mitochondrial membrane potential

Mitochondrial membrane potential was determined using JC-1 mitochondrial membrane potential assay kit (Cayman Chemical) according to the manufacturer's instructions. A fluorescent plate reader (BioTeK, VT, USA) was used, and red (excitation: 535 nm, emission: 595 nm) and green (excitation: 485 nm, emission: 535 nm) fluorescence was determined. Results are expressed as the ratio of red: green fluorescence.

### Statistical analysis

The data are expressed as the mean ± SD. Differences between groups were evaluated for significance by one way ANOVA analysis combined with Bonferroni post hoc test. All tests were performed using SigmaStat v3.5 (Systat-Software, Erkrath, Germany). A p-value below 0.05 was considered statistically significant.
